# Role of myocardial perfusion single photon emission computed tomography in pediatric cardiology practice

**DOI:** 10.4103/0974-2069.58314

**Published:** 2009

**Authors:** P Shanmuga Sundaram, S Padma

**Affiliations:** Department of Nuclear Medicine & PET CT, Amrita Institute of Medical Sciences and Research Center, Kochi, Kerala, India

**Keywords:** Kawasaki disease, pediatric myocardial perfusion, scintigraphy

## Abstract

Diagnostic and prognostic power of myocardial perfusion imaging in patients with coronary artery disease has been demonstrated with planar imaging which was further improvised with addition of gated SPECT and newer Technetium labeled myocardial perfusion tracers like SestaMIBI, Tetrofosmin. Myocardial perfusion abnormalities at rest and after stress are considered to be the best predictors of cardiac event–free survival in adults with ischemic heart disease. This article highlights various myocardial perfusion imaging (MPI) radiopharmaceuticals, exercise procedures, pharmacological stress protocols, indications for MPI and myocardial perfusion patterns in children with some of the common congenital and acquired heart diseases.

## INTRODUCTION

Utility of nuclear medicine techniques in adult cardiovascular diseases is well established for evaluation of myocardial ischemia, risk assessment, and viability. However, not many centers practice pediatric nuclear cardiology because of less referrals, difficulties in stressing children, and inadequate exposure to image interpretation. Myocardial perfusion imaging (MPI) in children is an important modality for evaluation of myocardial ischemia and viability. Patients with Kawasaki disease (KD), anomalous origin of the left coronary artery from the pulmonary artery (ALCAPA), coronary ectasia, and complete transposition of the great arteries (TGA) after arterial switch operation (ASO) can be easily evaluated non-invasively by MPI. In addition, it is also useful in patients with primary or secondary cardiomyopathies to assess the extent of myocardial damage. Children presenting with right ventricular (RV) pressure and/or volume overload can also be assessed by MPI. The RV free wall is normally not visualized on MPI when the patient is at rest. Only when there is an underlying pressure overload affecting RV myocardium does the single photon emission computed tomography (SPECT) imaging reflect positively. Uptake of myocardial perfusion tracers like SestaMIBI (Methoxy Isobutyl Isonitrile) MIBI/Tl (Thallium chloride) in septum and RV free wall is directly proportional to pressure overload. An inverted D sign[1] is characteristic of RV pressure overload conditions when RV diastolic pressure exceeds left ventricular (LV) diastolic pressure, producing an apparent straightening of septum. The magnitude of relative MIBI myocardial uptake of right to the left ventricle closely correlates with RV peak systolic pressure as well as the ratio of right-to- left ventricular peak systolic pressure in patients with congenital heart disease. Patients presenting with pure volume overload do not significantly show increased RV uptake. Rabinovitch, *et al*.[2] have used Tl planar scans to visually differentiate four grades of RV pressure loads. The absence of an apparent RV in MPI indicates that the RV pressure is within normal limits. A minimal to definite visualization of the RV that is less than the LV corresponds to a mild to moderate increase (30-70 mmHg) in the RV pressure and equally dense right and left ventricles is suggestive of a moderate to severe increase (50-100 mmHg). Radionuclide first pass is another technique that can be used to accurately estimate the right and left ventricular ejection fractions. Certain cases of long-standing congenital cardiac defects associated with volume overload may become further complicated with LV dysfunction, which can be detected much earlier with excellent reproducibility by MPI. Therefore, MPI can be used not only to identify myocardium at risk but can also provide cardiac quantitative indices like stroke volume, end systolic, end diastolic volumes, peak filling rates, emptying rates, etc. to optimize therapy and also serve as a prognostic yard stick.

With the growth of specialized pediatric cardiology centers and surgical techniques, there is a need for greater awareness of newer imaging procedures such as MPI in the diagnosis and prognostication of various pediatric cardiac problems. MPI is a useful physiological imaging modality in evaluation of myocardial viability and ischemia in children with congenital and acquired cardiac diseases that have associated coronary lesions.

## RADIOPHARMACEUTICALS

Radiotracers used for myocardial perfusion SPECT imaging are ^201^Thallium (^201^Tl) and Technetium (^99m^Tc) based radiopharmaceuticals such as SestaMIBI and Tetrofosmin. ^201^Tl, a potassium analogue, is used extensively in identification of myocardial viability, hibernation, and ischemia. Myocardial uptake of thallium is proportional to regional blood flow and depends on the presence of an active sodium potassium ATPase pump in the myocyte to facilitate its transport intracellularly.[3]

However, ^99m^Tc-labeled tracers are found to be superior to ^201^Tl with regard to imaging physics, radiation safety, availability, and cost effectiveness. Mechanism of myocardial uptake for Technetium-labeled agents is by passive diffusion and is therefore proportional to regional blood flow. Unlike ^201^Tl, they do not have the property of redistribution, hence reinjection is necessary for ECG gated rest imaging.[4]

**Figure 1:**
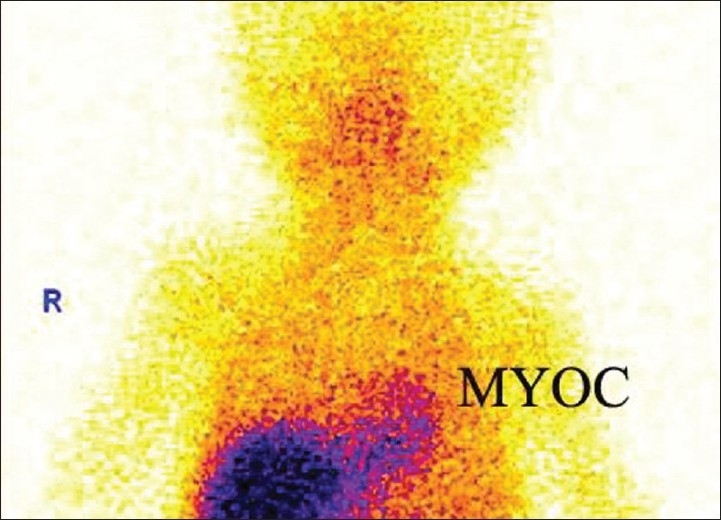
Post-stress planar image showing more than normal hepatic uptake of SestaMIBI in a 2-month-old male with congenital heart disease and thyroid agenesis

Technically, attenuation effects on LV tracer activity are almost negligible in pediatric patients (no inferior and anteroseptal wall attenuation effects). The main disadvantage of using ^99m^Tc-labeled tracers in children is their significant hepatic accumulation due to slow liver tracer clearance [Figure 1]. Vasodilators like dipyridamole or adenosine further enhance hepatic tracer accumulation in children undergoing MPI. To obviate this problem and minimize hepatic activity in children, a waiting period of at least 60 to 90 minutes post injection is recommended.

The minimum dosage of tracer to be administered intravenously in children is not well established so far.[5] However, there are studies reporting its safety in all age groups. Tc SestaMIBI is an ideal radiopharmaceutical as it produces less radiation exposure than Thallium. When used in a dose 0.200 mCi/Kg intravenously, SestaMIBI produces a radiation exposure of 0.49-0.69 rem, while Thallium at a dosage of 0.055 mCi/kg can lead to an exposure of 3.0 to 6.1 rem.[5] The reported absorbed radiation dose per equivalent injected dose of ^99m^Tc-labelled agents by the genitals in children is from 0.1-10% less than that of ^201^Tl.[6]

To evaluate myocardial ischemia, MPI is often conducted along with physical exercise or with pharmacologic agents both augmenting regional flow heterogeneity. Every pharmacological agent has a unique effect on the coronary vasculature and influences the distribution of the radiolabeled perfusion agent. Children and their parents must be counseled prior to undertaking any form of stress procedures for maximum motivation.

## STRESS PROCEDURE

Although exercise testing is more physiological, it is technically difficult to stress children on a treadmill or bicycle ergometer as they are basically designed for adults. An ergometer is preferable for exercising children because it is difficult to quantify the amount of work done on a treadmill apart from its high cost, noise production, and large size. Patients do not need to be ambulatory for ergometer testing. It is also difficult to obtain accurate blood pressure estimation while the child is on the treadmill because of the noise and motion artifacts. Disadvantages of ergometer exercise are that it needs more coordination to perform the test correctly and it is difficult to achieve maximum physical exertion unless the patient is pretrained on the bicycle. However, a treadmill is useful in children over 3 years where fast walking or running needs no special motivation or training.

The laboratory should be equipped with age and size-appropriate ergometers, emergency equipment, pediatric cuffs for the measurement of blood pressure, mouthpieces, and facemasks. It may be difficult to attain the maximum predicted heart rate in children, which is required to highlight abnormal coronary flow reserve. Serial evaluation might prove difficult in children, since exercise capacity significantly changes with physical growth during childhood. Symptom limited exercise protocols or modified pediatric protocols are followed with the aim of attaining the maximum possible heart rate. To increase workload, it is preferred to increase the grade of exercise rather than increasing the speed of the instrument.

Pharmacological stress is preferred in children for assessing the presence of coronary stenosis or serial evaluation of the same child while observing the progression of coronary stenosis. The incidence of significant adverse reaction to pharmacological stress in the pediatric population is unknown. In contrast, exercise stress might be more suitable and reflects the physiological changes while evaluating older children with exercise-related abnormalities (positive ischemic changes on exercise ECG), documented coronary arterial stenosis, or those under anti-ischemic medication and post coronary artery bypass graft (CABG) surgery patients. CABG in children is commonly done for ischemic complications of KD, congenital left main coronary ostial stenosis, iatrogenic coronary cameral fistula, ALCAPA, and single coronary artery traversing between the great arteries in post cardiac transplantation settings.[7] Asymptomatic, low-risk patients harboring myocardial ischemia are those with various heart diseases like atrial septal defect, ventricular septal defect, tetralogy of Fallot (prior to repair), Kawasaki disease without giant aneurysms, or patients with known coronary stenosis such as post-operative cases of D-TGA. Other patients such as those with Marfan syndrome, Takayasu arteritis, juvenile diabetes, systemic lupus erythematosus (SLE), nephritic syndromes, and treated cases of childhood lymphomas can also present with coronary insufficiency and atherosclerosis.[8,9]

Similarly asymptomatic patients post ALCAPA repair and ASO are also at low risk for inducing myocardial ischemia at a good workload. High-risk patients for provoking ischemia are those with pulmonary hypertension, dilated or restrictive cardiomyopathy in cardiac failure, and those with exercise- induced malignant arrthymias.

As per ACC/AHA/ESC 2006 guidelines (American College of Cardiology/American Heart Association/European Society of Cardiology)[10] for management of patients with ventricular arrhythmias and the prevention of sudden cardiac death, exercise testing with an imaging modality (echocardiography or MPI) is recommended to detect silent ischemia in patients with ventricular arrhythmias who have an intermediate probability of having coronary heart disease (CHD) by age, symptoms, and gender and in whom ECG assessment is less reliable because of digoxin use, left ventricular hypertrophy (LVH) , greater than 1-mm ST-segment depression at rest, Wolff-Parkinson-White (WPW) syndrome, or left bundle branch block (LBBB).

Similarly, patients with ventricular tachycardia (VT) and LV dysfunction due to prior myocardial infarction, cardiomypathy, bundle-branch reentry, and various forms of idiopathic VT planned for radiofrequency ablation also need to have documentation of ischemia with stress testing under strict supervision.

Endpoints for treadmill or ergometer exercise in children as part of MPI imaging as per the AHA guidelines are as follows:[11]

1. Decrease in ventricular rate with increasing workload associated with extreme fatigue, dizziness, or other symptoms suggestive of insufficient cardiac output
2. Failure of heart rate to increase with exercise, and extreme fatigue, dizziness, or other symptoms suggestive of insufficient cardiac output
3. Progressive fall in systolic blood pressure with increasing workload
4. Severe hypertension, >250 mmHg systolic or 125 mmHg diastolic, or blood pressure higher than can be measured by the laboratory equipment
5. Dyspnea that the patient finds intolerable
6. Symptomatic tachycardia that the patient finds intolerable
7. A progressive fall in oxygen saturation to <90% or a 10-point drop from resting saturation in a patient who is symptomatic
8. Presence of ≥3 mm flat or downward sloping ST-segment depression
9. Increasing ventricular ectopy with increasing workload, including a >3-beat run
10. Patient requests termination of the study

Consequently, MPI stress methods in children should be individualized and adjusted according to the clinical purpose and basal conditions.

## PHARMACOLOGICAL STRESS

Stressor agents and pharmacological stress procedure are the same as for adults. Pharmacologic stress agents fall into two categories: coronary vasodilators (e.g. adenosine, dipyridamole) and cardiac inotropic agents (e.g. dobutamine).

Usually, vasodilators are preferred for MPI. Vasodilators work directly on the coronary vessels and increase myocardial blood flow three to five times above the resting level, whereas inotropic agents work indirectly by increasing myocardial workload leading to an increase in coronary blood flow. Diagnostic accuracies for adenosine are similar to those of dipyridamole.

Adenosine promotes vasodilatation by activation of the A_2_ receptors in the vessels. It increases mean blood flow four to five times the baseline mean value. Dipyridamole inhibits the action of adenosine deaminase enzyme thereby inhibiting degradation of endogenously produced adenosine. It also blocks cellular reuptake of adenosine elevating endogenous adenosine causing vasodilation. Dipyridamole increases mean blood flow approximately three to four times of the baseline value.

Dobutamine is a synthetic catecholamine with a plasma half-life of 2 minutes that acts through the alpha-1 and beta-2 adrenoreceptors. Dobutamine mimics exercise physiology because it significantly increases the heart rate and blood pressure at doses greater than 20 μg/kg per minute. It artificially increases the cardiac output and myocardial oxygen consumption and therefore does not result in normal metabolic response to exercise.

## DOSAGES

Adenosine is infused at a dose of 140 μg/kg body weight per minute by an infusion pump for 4 to 6 minutes (total amount = 0.56 to 0.84 mg/kg). Abbreviated adenosine (3 to 4 minutes) protocols are also available.[12] Similarly, IV dipyridamole is infused over the same time period at a dose of 0.6 mg/kg per minute. A dose of 3 to 5 mg/kg of aminophylline given intravenously is the antidote for adverse effects due to dipyridamole.

Dobutamine is administered intravenously in gradations with a starting dose of 10 to a maximal dose of 50 μg/kg body weight per minute in 3- to 5-minute stages. Atropine (0.01 mg/kg up to 0.25 mg/kg aliquots given every 1 to 2 minutes up to a maximum dose of 1 mg) is also used to enhance the heart rate, if required. In children, a dose of dobutamine of 50 μg/kg per minute is usually necessary to achieve the target heart rate. Atropine is needed in approximately two-thirds of patients if the target heart rate is not achieved by dobutamine alone. Esmolol (10 mg/mL dilution) at a dose of 0.5 mg/kg is the antidote used to reverse the side effect or development of ischemia during dobutamine infusion.[13,14]

## CONTRAINDICATIONS

Children with bronchial asthma and heart blocks are contraindicated for vasodilator stress MPI (i.e. adenosine and dipyridamole stress).

Symptomatic infants and young children with severe congenital abnormalities of the left main coronary artery, ALCAPA, and ostial atresia of the left coronary artery are also not normally stressed.

## PATIENT PREPARATION

Motionless acquisition remains the primary concern during a myocardial perfusion SPECT imaging. This can be achieved with intravenous midazolam (0.1 mg/ kg body weight; maximum dose up to 0.5 mg/kg body weight) or pentobarbital in children younger than 5 years of age. Other conventional patient preparatory orders such as securing a patient IV line, 4 hours fasting prior to the test, and withholding beta-blockers and vasodilators remain the same as for adults.

## IMAGE ACQUISITION AND DISPLAY

SPECT images of myocardium are generally acquired using a 180-degree rotation from the right anterior oblique to the left posterior oblique projection. A dual-head variable angle gamma camera is ideal for dedicated nuclear cardiology centers. After reconstruction of the transaxial plane, the long axis of the heart is defined and for visual analysis, images are displayed in serial slices at 90-degree angles relative to the long axis of the heart. This allows for an optimal display of the ventricular chambers and perfusion abnormalities to specific coronary arteries can be identified. Many types of softwares have been developed for the quantitative analysis to assist in interpretation.

## INDICATIONS OF MYOCARDIAL PERFUSION SPECT IN CHILDREN

- Kawasaki disease
- Tetralogy of Fallot repair
- ALCAPA
- Post arterial switch operation
- Cardiomyopathies - hypertrophic cardiomyopathy, cardiomyopathy in Duchenne progressive muscular dystrophy
- Cardiac transplant
- Others: Estimation of right ventricular pressure overload, morphological RV as systemic ventricle

It is imperative to know that the normal distribution pattern of myocardial perfusion tracers in the LV is substantially different between children and adults. In children, the anterolateral segment of LV shows the least tracer uptake.[5] There can be anteroapical artifacts in adolescent females as a result of breast attenuation [Figure 2a and b]. Obese children may show reduced tracer uptake in the lateral segment of the LV. These artifacts can be easily identified in the stress and rest gated SPECT images as fixed defects with normal wall motion and good wall thickening.

**Figure 2a:**
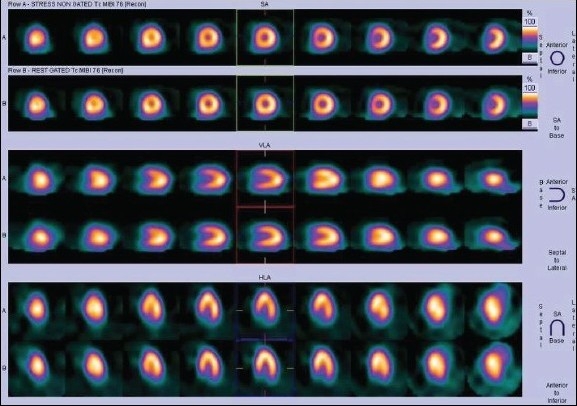
^99m^Tc MIBI normal stress myocardial perfusion single photon emission computed tomography

**Figure 2b:**
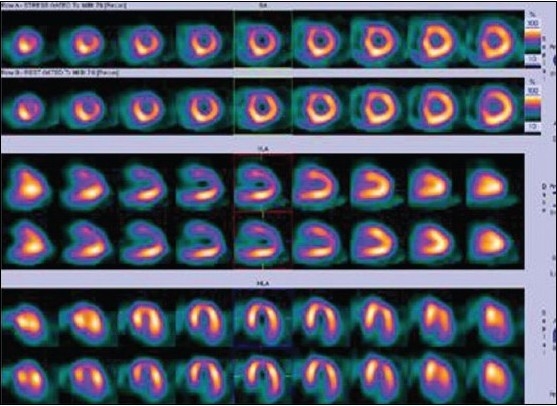
Mammary gland attenuation artifact in an adolescent patient - fixed perfusion defect in anterior segment

### Kawasaki disease

**Figure 3:**
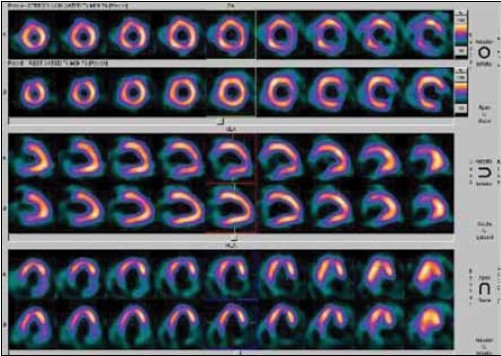
Stress MPI images of a 6-year-old male with a large lateral wall LV aneurysm. Stress SPECT images show moderate reversible perfusion defects in apex and inferior segments

**Figure 4:**
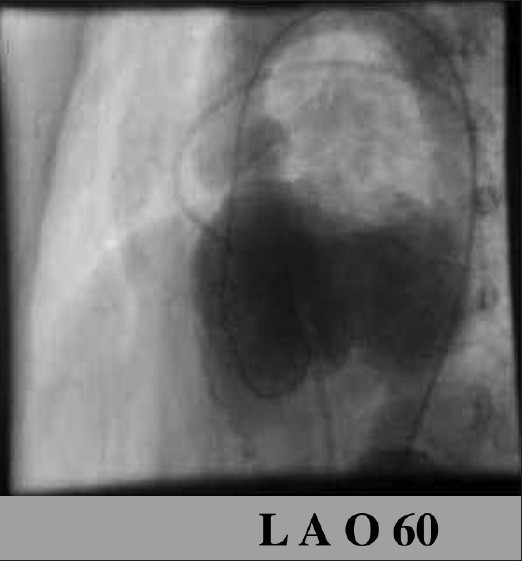
Left ventricular angiogram images of a 6-year-old male with a large lateral wall LV aneurysm.

MPI has mainly been utilized and studied extensively in pediatric cardiological practice for detecting myocardial ischemia in children with KD.[13,14] Sudden cardiac death may occur as a result of myocardial infarction in patients with extensive coronary arterial involvement. However, majority are generally asymptomatic despite significant risk for myocardial infarction.[13,15] Therefore, it is imperative to follow-up and detect coronary artery involvement in these patients. Literature reviews show that when studied by echocardiography, 20-25% of untreated children develop cardiovascular sequelae ranging from asymptomatic coronary artery ectasia or aneurysm formation to giant coronary artery aneurysms with thrombosis, myocardial infarction, LV aneurysm [Figures 3 and 4] and sudden death. Even today, 20% of untreated patients develop coronary artery aneurysms.[13]

Stress MPI is useful in children with KD to study the appearance or aggravation of myocardial ischemia and assess the success of coronary revascularisation. Either a pharmacological or exercise MPI can detect angiographically proven coronary stenosis with 70-90% sensitivity.[14,15] It can identify single coronary vessel stenosis in KD more accurately than an exercise stress ECG. Stress MPI also can be used for cardiac risk stratification in these cases.[14,15]

The presence of reversible perfusion defect on dipyridamole-stress MPI is a powerful predictor of cardiac events during the chronic stage of KD[15] [Figure 5a and 5b].

**Figure 5a:**
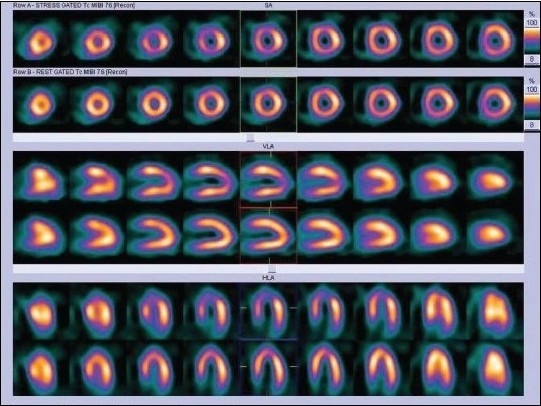
Same day stress - rest MIBI SPECT images (displayed in two different color scales) of a 12-year-old patient with Kawasaki disease showing mild reversible ischemia in the apex, anteroapical, and septal segments of the LV

**Figure 5b:**
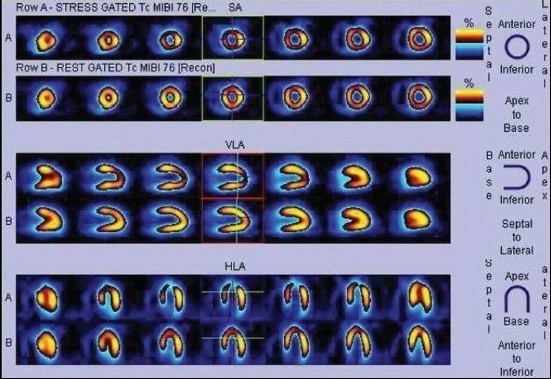
Same day stress - rest MIBI SPECT images (displayed in two different color scales) of a 12-year-old patient with Kawasaki disease showing mild reversible ischemia in the apex, anteroapical, and septal segments of the LV

### MPI in congenital and acquired heart diseases

#### Tetralogy of Fallot

**Figure 6:**
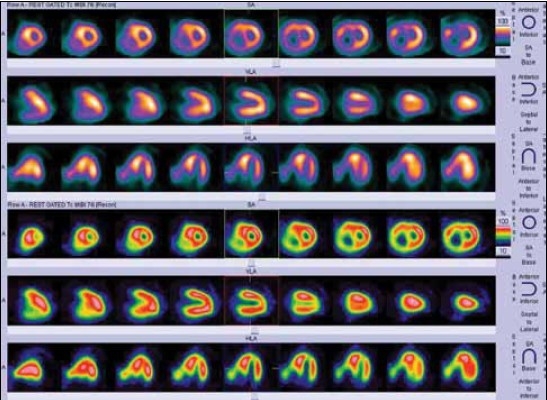
Rest SPECT images of an uncorrected tetrology of Fallot patient with biventricular dysfunction. Images show right ventricular enlargement, hypertrophy, and associated septal hypertrophy. There are no perfusion defects in LV myocardial segments at rest

In tetralogy of Fallot, corrective surgery is usually performed above six months of age. Correction involves closure of the ventricular septal defect with a patch and relieving the pulmonary stenosis. An increasing number of patients have residual abnormalities of ventricular function and cardiovascular performance after right ventricular outflow tract (RVOT) repair. A myocardial perfusion SPECT may be useful in identifying residual or inducible ischemia, regional ejection fraction estimation, wall motion abnormality, and residual hypertrophy [Figure 6].

### Anomalous origin of the left coronary artery to pulmonary artery

With the recent availability of MDCT, conditions such as ALCAPA can be best diagnosed using MDCT. Practical difficulties of breath holding in newborns, infants, and children can be overcome with MPI where it can be easily performed with less radiation and better compliance.

Patients with ALCAPA usually show severe perfusion defects at rest. Caution must be exerted to avoid inducing myocardial ischemia unnecessarily in these patients, which may be of less clinical relevance and may cause life threatening sudden cardiac events. Therefore, pre-surgical myocardial viability determined by a rest MPI might be adequate to determine if a patient will benefit from any revascularization procedure. Patients showing 50% and more MIBI or Thallium uptake in a LV segment is said to be viable.[3]

**Figure 7:**
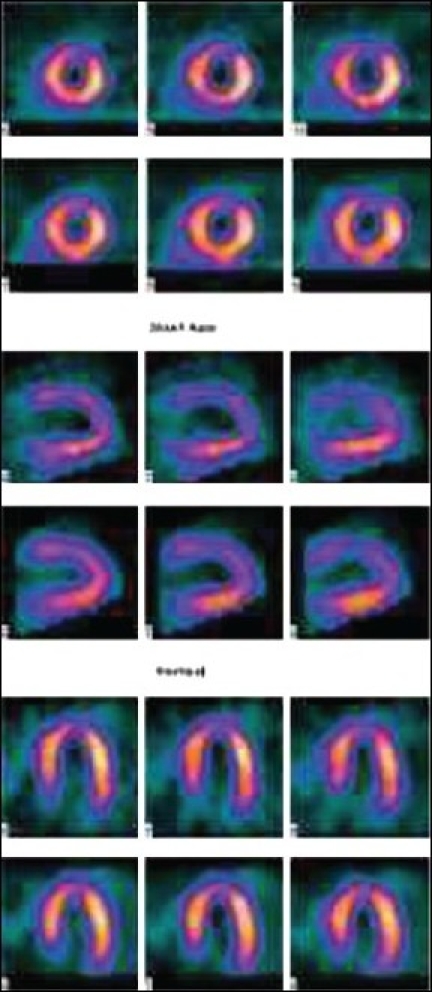
Stress and rest single photon emission computed tomography images of an asymptomatic adolescent boy with uncorrected ALCAPA syndrome showing left anterior descending artery territory ischemia in the background of an old infarct

Stress MPI is also useful in treated or fairly asymptomatic older children and adults with ALCAPA as a follow-up to assess any fresh inducible myocardial ischemia [Figure 7]. Such patients can have localized infarction and an almost entirely collateral circulation-dependent perfusion of the LV that results in poor global LV function. If untreated, survival beyond infancy is uncommon in this congenital abnormality because of severe left heart failure. Revascularization, however, brings about functional recovery with a good clinical outcome.

For infants with ALCAPA syndrome, because the left main coronary artery territory is perfused retrogradely by collateral circulation from the right coronary artery, extensive perfusion abnormalities are more frequent at apical and inferior walls. Once reimplantation of the left coronary artery into the aorta is performed, studies indicate slow but significant improvement in myocardial dysfunction within the next year.[16] Scintigraphy wise fixed or reversible perfusion abnormalities indicating either a scarred or ischemic myocardium can persist in a few cases even after successful coronary reimplantation. This can be related to the presence of preoperative myocardial injury and persistent post surgical microvascular dysfunction. Hurwitz, *et al*.[17] found a preoperative left ventricular ejection fraction of 0.37 ± 0.16 increasing to 0.67 ± 0.07 after surgery in infants and children with ALCAPA. In such instances, MPI is helpful in evaluating the myocardial viability when considering surgical options for ALCAPA.

MPI is also useful in differentiating ALCAPA from congestive cardiomyopathy in infants. Patients with little collateralization become symptomatic early in life with cardiac failure due to ischemic heart lesions. Scintigraphic findings are characteristic in such patients with ALCAPA and are localized to high lateral, anterior, and lateral segments[16,18] while patients with cardiomyopathy show apparent perfusion defects in a grossly dilated LV myocardium with severe hypokinesia in gated images. Despite these diagnostic patterns, a review of literature shows that MPI has been sparingly used in the evaluation of ALCAPA. Although conventional echocardiography and magnetic resonance imaging provide information on LV dysfunction and anatomical abnormalities, MPI still holds its place due to its physiological basis of testing, high reproducibility, and no interobsever variability. As per ACC/AHA 2008 Guidelines,[19] surveillance with echocardiography and noninvasive ischemia provocation testing is necessary every 3 to 5 years for patients after the repair of ALCAPA. This is due to increased awareness of residual myocardial and valvular abnormalities despite an adequate repair.

### Complete transposition of the great arteries after arterial switch operation

Surgical management of D-TGA has evolved over several decades.[20‐22] Definitive anatomical correction, however, was not described until 1975 with the advent of Jatene’s ASO.[23] Anatomical correction was intended to avoid the long-term complications of a systemic RV and the complex atrial arrhythmias inherent to the Mustard and Senning operations.[24,25]

The ASO is the current surgical procedure of choice in newborns and infants with transposition of arteries and involves mobilization and reimplantation of coronary arteries into the neo-aorta.[26] Thus, the long-term success of this procedure depends on continued patency of coronary arteries, which can be addressed by MPI.

Several investigators have used MPI to study any variations in myocardial perfusion in these children after surgery.[26] As in KD, proximal obstruction of the translocated coronary artery can be detected by stress-induced perfusion defects on MPI. Weindling, *et al*.[27] also found reversible perfusion abnormalities in patients after ASO most commonly in the apical, lateral free wall of the left ventricle corresponding to the distal territories of the left coronary artery. Perfusion scan abnormalities after ASO reflect not only proximal coronary obstruction but also arteriolar or capillary pathophysiology with abnormal vasomotor tone at rest or in response to exercise or pharmacological stimuli. All of these effects might be exacerbated and become apparent as perfusion defects in the territories of the hypoplastic distal left coronary artery in patients after ASO.

### Cardiomyopathies

**Figure 8:**
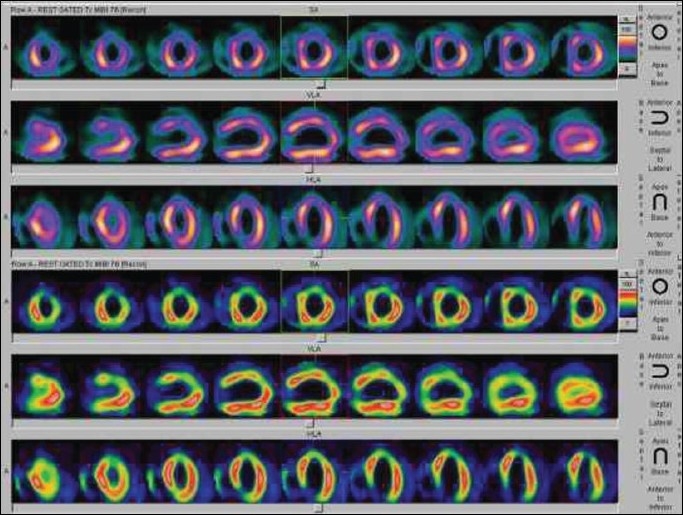
Rest gated MIBI SPECT images in a patient with dilated cardiomyopathy. Apparent perfusion defects seen in the apex and inferior segments of the LV myocardium

Recent evidence shows that in all new cases of dilated or hypertrophic cardiomyopathy, PET or scintigraphy should be performed to exclude coronary artery anomalies in children.[28‐31] Compression of coronary arteries and their septal branches is common in children with hypertrophic cardiomyopathy and is related to the magnitude of left ventricular hypertrophy. MPI may show apparent perfusion defects in severely hypokinetic myocardial segments in patients with dilated cardiomyopathy [Figure 8].

#### Hypertrophic cardiomyopathy

Children diagnosed with hypertrophic cardiomyopathy (HCM) are at a higher risk of sudden death, which is usually due to inducible myocardial ischemia rather than ventricular arrhythmias. Theodoro, *et al*.[32] from the Mayo Clinic reported that 96% of young patients (between 2 months and 20 years of age; mean age = 1.2 years) in their series had significant improvement in preoperative symptoms after surgery. An exercise stress MPI done in children with HCM can be very informative in those undergoing extended septal myectomy, which is a safe and effective method of relieving cardiac symptoms and left ventricular outflow tract obstruction in pediatric patients with severe hypertrophic obstructive cardiomyopathy unresponsive to medical management. Similarly, patients undergoing enlargement of their left ventricular outflow tract by Konno or a modification of the Konno procedure can also benefit from MPI. As the final step of the Ross Konno procedure involves the right ventricular outflow tract reconstruction with the pulmonary autograft, there are possibilities of allograft kinking, coronary artery compression, or anastomotic stricture due to the location of pulmonary bifurcation posterior to the neoaorta. Similarly, because of the proximity of the septal perforating branches of the left anterior descending (LAD) artery to the suture line, there are chances of septal perfusion getting compromised. Therefore, MPI can be used in the follow-up period to assess early diastolic dysfunction and LAD ischemia. There is, however, no data on the use of MPI especially in this subset of patients on literature survey.

In HCM, there can be reversible perfusion abnormalities in anterior, septal, apical regions, or subendocardial ischemia shown as transient stress induced dilatation of the left ventricular cavity.[33] The mechanisms of ischemia in these patients abnormality of intramural small vessels, abnormal myocellular architecture, or myocardial hypertrophy.

#### Cardiomyopathy in Duchenne muscular dystrophy

Duchenne muscular dystrophy (DMD) is associated with myocardial degeneration and fibrosis. The distribution of myocardial lesions is segmental and initially located throughout the basal inferior and contiguous lateral walls of the LV.[34] In later stages of this disease, there is reduced perfusion in midinferior, apical, and anterior segments of the LV that corresponds to severe transmural fibrosis and fatty infiltration. Positron emission tomography (PET) studies show increased glucose utilization (perfusion metabolism mismatch) mainly in the posterobasal and posterolateral walls of LV.[34]

### Myocardial perfusion imaging in cardiac transplant recepients

Today, pediatric heart transplantation covers an age range from the fetus listed for transplant at 36 weeks to the late adolescent. Diagnoses range in complexity from cardiomyopathy to the most complex of congenital heart malformations. The five-year survival rate for cardiac transplants is greater than 70%. As per Wigfield, *et al*.[35] the actuarial survival at 2 years is 87% and 82% at 10 years. MPI is crucial in the presurgical workup of cardiac transplant patients to identify any physiologically significant coronary stenosis.

In post-transplant evaluations, there are conflicting reports of poor sensitivity of MPI to detect transplant vasculopathy (TV),[36] which is one of the most serious complications. It is an immune-mediated endothelial injury producing diffuse intimal concentric hyperplasia with intimal proliferation of vessels culminating in fibrosis.[37] After recognizing alloantigens of graft, the host immune system T lymphocytes release cytokines that recruit more inflammatory cells, modulate antigens expression on allograft vascular cells, and regulate growth of smooth muscle cells. Once initiated, this process continues progressively leading to obstructive coronary vasculopathy. Hypertension, hypercholesterolemia, hypertriglyceridemia, and viral infections (Cytomegalovirus) can accelerate TV. Pathologically rejection produces myocyte necrosis and apoptosis associated with interstitial mononuclear cell infiltration. Nuclear imaging can be used for non-invasive detection of these pathological entities.

Radiopharmaceuticals used in post cardiac transplant evaluations are ^99m^Tc Annexin V and ^111^In Pentetreotide. Although both agents are used to identify graft rejections, their mechanisms of action are different. ^99m^Tc labeled Annexin V imaging[38] is used to identify graft rejection non-invasively. Annexin V is a medium-sized endogenous human protein tagged with ^99m^Tc. This has a high Ca^2+^ dependent affinity towards phosphatidyl serine (PS). Apoptosis or cell death exposes a phospholipid, PS that is normally confined to the inner leaflet of the cell membrane bilayer. Annexin’s high affinity for binding to PS is the basis for apoptosis imaging. Somatostatin analogues like^111^In Pentetreotide[39] are being propagated to target activated lymphocytes involved during acute cardiac rejection for imaging. This can predict rejection at least 1 week prior to an endomyocardial biospy. ^99m^Tc Annexin and ^111^In monoclonal antimyosin imaging hold promise in cardiac transplant patients but need more sample studies.

### Other utilities of myocardial perfusion imaging

#### Estimation of right ventricular pressure overload by myocardial perfusion imaging

Normally, the RV is not visualized in MPI basically because of higher LV muscle mass. However, myocardial uptake of perfusion tracers by the RV wall is increased and marked in patients with pressure with or without volume overload of the RV.[2] With regard to scintigraphy, the degree of RV wall uptake is proportional to RV pressure. Increased tracer uptake in RV and a peak count ratio of RV to LV over 0.45 has been advocated for RV pressure overload. Interventricular septal flattening (“inverted D”) as a scintigraphic criterion of RV pressure overload has also been reported by Movahed, *et al*.[1]

#### Morphological right ventricle as a systemic ventricle

Surgery for congenital heart disease may be divided into two types: Univentricular or biventricular. Biventricular repair encompasses situations wherein both ventricles are separately used to support the systemic and pulmonary circuits, such as repair of ventricular septal defect or tetralogy of Fallot. Univentricular palliation, also known as Fontan or Fontan-type repair[40] may be necessary for patients with tricuspid atresia, double inlet ventricle, or hypoplastic right/left heart syndromes when the heart cannot be septated.

Therefore, in both these cases where morphological RV functions as a systemic ventricle, MPI can be performed to assess perfusion defects. Both reversible and fixed perfusion defects are observed more commonly in anterior, inferior, and septal walls rather than in lateral walls. The extent of fixed defects corresponds to the degree of depressed global RV function[41] and reversible perfusion defects reflect an inability of the right coronary arterial system to supply the systemic hypertrophied RV. Studies by Kondo, *et al*.[42] using ^201^Tl and ^123^I-BMIPP (Iodinated fatty acids) have been undertaken in patients with a single RV to show that the myocardium is normally perfused and is hypertrophied at rest but metabolically compromised at apico-anterior regions with regional contractile dysfunction. Myocardial SPECT which highlights this contractile dysfunction in cyanotic heart disease is primarily linked to impaired free fatty acid metabolism rather than to a myocardial scar, an information which cannot be obtained from other imaging modalities. This also has prognostic implication in patient management as it signifies an ongoing microvascular or myocardial derrangement thus providing an opportunity for intracardiac repair.

## MYOCARDIAL PERFUSION IMAGING AND OTHER CORRELATIVE IMAGING TECHNIQUES

Various imaging techniques are used to assess cardiac status. Imaging of the heart has been limited to cardiac catheterization, echocardiography, and nuclear medicine imaging. With new advances in both hardware and software in the last two decades, there is now a major change in the role of cross sectional imaging, i.e., CT and MRI, in the assessment of cardiac disease.

### Echocardiogram

Echocardiograms are universally available, cheap but operator dependent, and fail in cases of poor Echo window. A dobutamine stress echocardiography (DSE) exhibits a comparable accuracy with respect to MPI for diagnosing coronary artery disease, localizing coronary artery stenosis, and detecting regional myocardial abnormalities.[43] The wall motion (WM) score index may be useful for evaluating the myocardial area at risk. When compared with myocardial perfusion, WM responses to dobutamine may underestimate the severity of coronary artery disease (CAD) that exists or fail to detect CAD if a critical level of demand stress is not achieved. In this regard, real-time echocardiography has been demonstrated to improve the sensitivity and accuracy of DSE by simultaneously assessing myocardial perfusion along with WM.[44] The mechanism for the higher sensitivity of MPI over WM appears to be related to the cascade of pathophysiological events during demand ischemia, in which myocardial perfusion defects precede WM abnormalities during incremental dobutamine infusion as studied by Jeane, *et al*.[44] As the target heart rate during DSE is defined by patient age, test endpoints may not be always achieved.

A multislice CT now provides retrospective cardiac gating and the entire procedure could be accomplished in one breath hold. With excellent spatial (0.625 mm) and temporal resolution (0.5 sec),[45] CT imaging of the cardiovascular system allows assessment of the fine details without cardiac and breathing related motion artifact. Multiplanar reconstruction and 3D reconstruction enable better appreciation of the anatomy, which proves to be particularly useful in preoperative planning and assessment. The major advantage of multislice CT regarding estimation of left ventricular parameters is the shorter examination time per patient. However, its limitations are lower temporal resolution, the need for nephrotoxic contrast material and substantial radiation doses up to 13 mSv[46] delivered during a single CT scan. The administration of β-blockers in some patients to control the heart rate before CT is one of the relative limitations.

### Myocardial perfusion imaging

For CAD evaluation, physiological significance of a known coronary lesion, collateralization of vessels, revascularization strategies (CAGB vs PTCA, single versus dual stenting/CABG) can only be answered by stress gated MPI/PET studies with excellent reproducibility. With the availability of SPECT-CT scanners, both MPI and multislice CT information obtained in the same sitting can be overlaid to provide better roadmaps to the cardiologist. DSE and stress thallium-201 MPI were compared for detecting coronary artery disease in 120 consecutive patients who underwent concomitant quantitative coronary angiography.[43] Both tests showed 81% agreement in all 120 patients. The overall sensitivity of DSE and MPI for the detection of coronary artery disease was 85% and 89%, and the specificity was 93% and 85%, respectively.

The DSE has been reported to be a safe and feasible pharmacological method for the evaluation of patients with suspected CAD. But in children with variable heart rates, MPI may become abnormal while WM is still normal, resulting in both higher sensitivity and diagnostic accuracy of MPI over WM analysis for detecting CAD.[43] Another possibility is that WM abnormalities induced by dobutamine could be missed when using the slower frame rates (25-30 Hz) utilized with real-time perfusion imaging. However, the increased ability to visualize the endocardial border with ultrasound contrast aids in the detection of dobutamine-induced hypokinesis.

## MRI

Standard MRI techniques do not allow real-time imaging for on-line assessment of a pharmacologically-induced myocardial contraction reserve, although this may become possible in the future with improved echo-planar techniques. Patients with a pacemaker and implants are contraindications for MRI. Moreover, quantitative assessment of wall thickness at rest and after dobutamine infusion is time-consuming and difficult to use for routine clinical purposes. However, faster imaging techniques[47] used in conjunction with semi-automated analysis of wall thickening changes between rest and dobutamine studies[48] may soon facilitate and promote the clinical use of MRI.

## PET-CT

Gold standard investigation in the assessment of myocardial viability as of now is FDG PET. Preserved glycolytic activity as estimated by regional FDG uptake in myocardial regions with impaired function has been reported to be accurate for the differentiation of viable myocardial tissue from scar. Myocardial perfusion imaging (MPI) with SPECT and flouro deoxy glucose (FDG) PET are well suited to provide all this information apart from quantification of these parameters. FDG-PET is superior to all existing imaging modalities as it provides metabolic status of myocardium prior to CABG. Although preservation of end-diastolic wall thickness as assessed by MRI in akinetic myocardial regions was proposed as an indicator of viable myocardium[49] studies have challenged the reliability of myocardial morphology as assessed by MRI at rest for the identification of viable myocardium[50] because metabolic activity can be observed in regions with significantly reduced end-diastolic wall thickness and no wall thickening.

## CONCLUSION

There is no investigation that can provide all information without fallacy. Today we are privileged to have multiple imaging modalities and fusion imaging at an affordable cost with 2D and 3D volume rendering reconstruction advancements. It is for us to choose the right blend of anatomical and physiological imaging techniques to optimize patient care.
